# Public health law coverage in support of the health-related sustainable development goals (SDGs) among 33 Western Pacific countries

**DOI:** 10.1186/s12992-019-0472-z

**Published:** 2019-04-11

**Authors:** Yuri Lee, So Yoon Kim

**Affiliations:** 1 Department of Global Health, Graduate School of Public Health, Yonsei University, #410, Administration B/D, Yonsei University Health System, 50-1, Yonsei-Ro, Seodaemun-gu, Seoul, 03722 Republic of Korea; 2 Asian Institute for Bioethics and Health Law (WHO Collaborating Centre for Health Law and Bioethics), College of Medicine, Yonsei University, Seoul, Republic of Korea

**Keywords:** Public health law, Sustainable development goals, Western pacific countries

## Abstract

**Background:**

A resilient health system is inevitable in attaining the health-related Sustainable Development Goals (SDGs). One way of strengthening health systems is improving the coverage of public health laws for better health governance. The aim of this study is to describe the public health law situation in the Western Pacific Region and analyse the association of public health law coverage with health-related SDGs statistics.

**Methods:**

A total of 33 Western Pacific countries were selected and analysed using a multi-group ecological study design. Public health law coverage was measured from April 2013 to October 2016 based on the public health law coverage module in the ‘Tool to Assess Health Law’ developed by the WHO Western Pacific Regional Office and Asian Institute for Bioethics and Health Law of Yonsei University. The health-related SDGs status were examined using health statistics data from World Health Statistics 2017 and 2018 by WHO and SDGs index scores of previous research.

**Results:**

Countries with high public health law coverage were Vietnam, Republic of Korea, Hong Kong, and Singapore. Low coverage countries were mainly Pacific Island countries. High public health law coverage issues were health care organisation, communicable diseases, and substance abuse, whereas those of low coverage were human reproduction, family health, and oral health. Public health law coverage was associated with health-related SDGs statistics such as life expectancy at birth (r = 0.47, *p* = 0.03), health life expectancy at birth (r = 0.47, *p* = 0.04), health-related SDGs index (r = 0.43, *p* = 0.05). Among the SDG 3 indicators, maternal mortality ratio (r = − 0.53, *p* = 0.01), neonatal mortality rate (r = − 0.44, *p* = 0.02), new HIV infections (r = 0.78, *p* = 0.04), total alcohol consumption (r = 0.45, *p* = 0.02), adolescent birth rate (r = − 0.40, *p* = 0.04), UHC service coverage index (r = 0.50, *p* = 0.02), and IHR average core capacity score (r = 0.54, *p* = 0.004) were statistically meaningful. However, there was no association of public health law coverage with health statistics in other SDGs.

**Conclusions:**

This study proved the importance of public health law in supporting the attainment of health-related SDGs. These results should be used as the basis for review and action at country level in improving public health law for better health systems, consequently achieving health-related SDGs.

## Background

Global society has already entered into the era of the United Nations (UN) Sustainable Development Goals (SDGs) which has address global challenges from 2016 to 2030. Among the 17 SDGs, health issues are covered in SDG 3 of good health and well-being articulated as ‘ensuring healthy lives and promoting well-being for all at all ages’. This includes 9 targets and 4 means of implementation. It contains several health targets for accelerating the Millennium Development Goals (MDGs) and includes other issues such as non-communicable diseases, substance abuse, traffic accidents, reproductive health, universal health coverage (UHC), and environmental pollution and contamination, which are excluded in the MDGs [1]. Health issues are also indirectly covered in the other 16 SDGs and targets [2]. This means that SDGs highlight organic connections between various health factors and discuss health issues more comprehensively than MDGs [3]. The MDGs mainly concentrate on a vertical approach as opposed to the SDGs that focus on a horizontal approach; a systematic approach that not only tackles single diseases or intervention strategies but also the overall health problems on a wide front and on a long-term basis [4]. From this point of view, the strengthening of health systems has become more important in the SDGs era. The most important challenge is how to globally achieve SDGs [5]. In fact, countries face challenges in the attainment of advanced health-related SDGs [6].

World Health Statistics 2017 by World Health Organization (WHO) clearly demonstrate how we can achieve the health-related SDGs [7]. Considering the health-related SDGs’ points of impact, strengthening health systems could be the input and UHC an output from the logical model. The SDGs health-related targets cannot be achieved without making substantial progress on strengthening the health systems so as to deliver effective and affordable services.

Strengthening the health systems requires a co-ordinated approach involving improved health governance [1]. Further, public health law is essential in the process of better health governance [8]. In this regard, public health law plays a crucial role in achievement of the health-related SDGs and requires an understanding of their interaction with other modes of action to influence health promotion and protection. One of the effective ways for resilient governance is establishing good public health law systems in countries [9]. Public health law is central to establishing a health system through defining public health objectives, allocating responsibility for policy making, planning and standard setting as well as determining the roles and responsibilities of relevant government agencies [10]. It also facilitates the co-ordination and regulation of governmental and non-governmental health related activities.

Health system is closely associated with a significant amount of health legislations as well. For example, the medical services act or nursing law is related to the health service delivery factor. Medical products and technology also apply relevant public health laws such as the food and drug administration, blood safety, essential drug list, and poisons acts etc. In order to achieve the health-related SDGs, it is essential to strengthen the health system [11] and other relevant health legislations. The mechanism of law can contribute to attaining SDGs through strengthening the health system. In other words, for successful mainstreaming of the SDGs into national health policy, public health law plays an important role in making a commitment.

Since the late 1990s, there have been floating public health law researches that explore how laws influence environments, behaviours, and beliefs on health, therefore creating an impact in the prevention and management of diseases as well as injuries in a population [10]. One main stream of public health law is legal epidemiology which focuses on the law’s health effects, particularly evaluating the health status legal interventions [12, 13]. As the concept of evidence-based public health practice advances, the importance of this kind of research has increased. However, this is extremely rare. Besides, it is more difficult to find an inter-country rather than intra-country unit of analysis.

The aim of this study is to describe the public health law situation in the Western Pacific Region in providing a broad landscape and analysis of the association of public health law coverage with the statistics of health-related SDGs. Specifically, the scope of this study is to assess the current status of public health law in the relevant countries as well as specific subjects such as finding the differences in public health law coverage by socio-economic status, basic characteristics, and legal situations in countries. Further, the scope includes confirming the association of public health law coverage with health indicators in SDGs. Thus, the study intends to stress the importance of strengthening public health law in countries and suggest future policy strategies and actions towards achieving the SDGs.

## Methods

### Project design

Since May 2011, the WHO Western Pacific Regional Office (WHO/WPRO) and the Asian Institute for Bioethics and Health Law (AIBHL) of Yonsei University have been engaging in a long-term project to monitor the public health law situation among Member States. The purposes were to develop an analysis tool for assessing country-level public health laws and to conduct in-country analysis using the tool for better health systems. Through expert consultation meetings [14, 15], the ‘Tool to Assess Health Law’ was developed. It consists of ‘yes’ or ‘no’ questions assessing whether a country has enacted laws in a given area [16].

Data on domestic public health laws including constitutions, primary, and subsidiary legislations were collected. A survey was also applied using the tool in the Western Pacific Region [17]. Application of the tool has been completed in 36 of the 37 countries. China was excluded because we could not find an appropriate local researcher. The collected data are accessible through the WHO/WPRO online library as well as online databases of public health laws in the official website of the AIBHL of Yonsei University. This study used some part of the assessment results on in-country analysis on public health law as the public health law coverage module.

### Study design

Using a multi-group ecological study design, this research attempts to describe the public health law situation in the Western Pacific Region and to analyse the association of public health law coverage with health-related SDGs indicators. A total of 33 countries were selected as study participants after excluding 3, French Polynesia, New Caledonia, and Wallis and Futuna, due to missing data. The 33 countries are American Samoa, Australia, Brunei Darussalam, Cambodia, Cook Islands, Fiji, Guam, Hong Kong, Japan, Kiribati, Lao People’s Democratic Republic, Macao, Malaysia, Marshall Islands, Federated States of Micronesia, Mongolia, Nauru, New Zealand, Niue, Northern Mariana Islands, Palau, Papua New Guinea, Philippines, Pitcairn Islands, Republic of Korea, Samoa, Singapore, Solomon Islands, Tokelau, Tonga, Tuvalu, Vanuatu, and Vietnam.

**Fig. 1:**
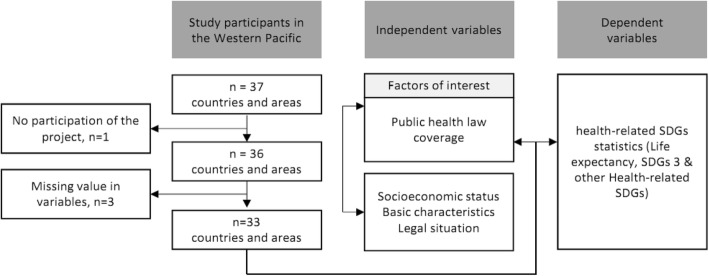
Study design frame

The general characteristics of study participants were examined and the extent of public health law coverage was identified in the countries and subjects. Differences of public health law coverage by socio-economic status, basic characteristics, and legal situation of the countries were described. Further, the relationship between public health law coverage and life expectancy, SDG index, health statistics in SDG 3, and other SDGs were analysed to prove the importance of public health law in attaining the health-related SDGs. The independent and dependent variables are public health law coverage and health status from health-related SDGs indicators, respectively as shown in Fig. 1.

### Data collection and extraction

Data collection on public health law was conducted from April 2013 to October 2016. In each country, local researchers with public health law expertise were nominated by the Ministry of Health (MOH). As a local researcher for the representing country, he or she conducted the two processes for completing the mission. They gathered data on public health laws from libraries, government archives, and web-based databases for filling the tool. Desk reviews were supplemented by consultation meetings with several experts in public health law and country policies. The expected consultants were WHO country office technical staff, central and local government officers, and public health or law specialists. We aggregated all country level data including legal systems, list of public health law by constitution and primary legislation, and existence of health law using questionnaires. Socioeconomic status and basic characteristics of countries were examined based on the UN classification [18].

Data of the health-related SDGs were from the World Health Statistics 2017 and 2018 by WHO [7, 19]. We extracted the statistics of the 33 countries among all the WHO Member States. There were two general indicators including life expectancy at birth and healthy life expectancy at birth which are not SDGs indicators but measure the overall health status. These are major health indicators, which means if the health-related SDGs are attained then life expectancy is also increased. We extracted 20 indicators which are included in SDG 3 and 12 indicators in other SDGs which are indirect health-related factors. Most of the statistics except the target 3.8 UHC indicators are from the World Health Statistics 2017 while UHC data are from World Health Statistics 2018. There was no consensus about what kinds of indicators should be monitored for UHC until 2017, explaining why the UHC data was put first in the World Health Statistics 2018.

Total SDGs index score data were from the 2017 report by the Sustainable Development Solution Network (SDSN), which provides a comprehensive set of county-level data for SDGs. The SDGs index score signifies a country’s position between the worst (0) and best (100) outcomes across the 17 SDGs [20]. The health-related SDG index was published by the Global Burden of Diseases, Injuries, and Risk Factor 2015 study (GBD) on SDGs collaborators, to systematically compile data for estimating the performance of the health-related SDGs indicators in 188 countries from 1990 to 2015 [21].

### Statistical analysis

Using quantitative analysis methods, the general characteristics of the countries were analysed by frequency and percentage. Public health law coverage of countries classified by subjects were also analysed by frequency and percentage, while the mean and standard deviation were demonstrated by sub-categories. Differences of public health law coverage by socio-economic status, basic characteristics, and legal situation of countries were analysed using the ANOVA and t-test. For examining the association of public health law coverage with health-related SDGs statistics, the chi-square test and simple regression analysis were applied. For all analyses, *p* ≤ 0.05 was considered statistically significant using the SAS statistical software package version 9.4 (SAS Institute Inc., Cary, NC. USA).

## Results

Table 1 shows the general characteristics of study participants. Among the 33 countries, there are 11 high-income, 8 upper middle-income, 10 lower middle-income, and 4 countries without UN data. There were no low-income countries. With regards to the poverty level, there were 6 least developed countries. Of the remaining 27, 20 were small developing islands. There are 24 sovereign states which cover 72.7% and 9 overseas territories and dependent regions from the United States of America, France, the United Kingdom, and New Zealand. Regarding the legal system, 21.2, 69.9, and 9% of the countries had civil, common, and combination law systems, respectively. Twenty countries and regions (60.6%) have health-related provisions in their constitutions. However, we could not establish the kind of provisions in the other countries. The average number of health-related primary legislations were 28.1, while 27.2, 57.5, and 15.5% represent countries with 0–20, 21–40, and countries with more than 41 primary health legislations, respectively. Mongolia had 68 primary health legislations, which is the highest among the participant countries.

**Table 1:** General characteristics of study participants

Variables	Categories	(*n* = 33)
		N (%)
Income	High-income country	11 (33.3)
	Upper middle-income country	8 (24.2)
	Lower middle-income country	10 (30.3)
	No data	4 (12.1)
Poverty	Least developed country	6 (18.1)
	Non-least developed country	27 (81.8)
Islands nations	Small islands developing states	20 (60.6)
	Others	13 (43.3)
Sovereign and dependent areas	Sovereign states	24 (72.7)
	Overseas territories and dependent areas	9 (27.2)
Legal system	Civil law	7 (21.2)
	Common law	23 (69.6)
	Combination law	3 (9.0)
Health Provision in constitution	Yes	20 (60.6)
	No	13 (43.3)
Number of Primary Health legislations	0–20	9 (27.2)
	21–40	19 (57.5)
	41 and over	5 (15.5)

**Fig. 2:**
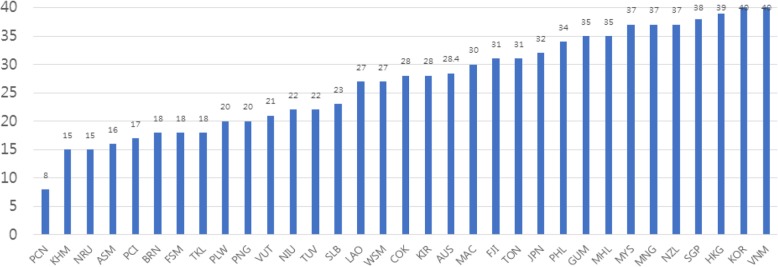
Public health law coverage by countries

Figure 2 demonstrates the public health law coverage by countries. The average number of ‘yes’ responses among the 33 countries was 26.9 out of the 40 questions, with a standard deviation of 8.8. Countries with high public health law coverage were Vietnam, Republic of Korea, Hong Kong, Singapore, New Zealand, Mongolia, and Malaysia. Countries with low public health law coverage were Pitcairn Islands, Cambodia, Nauru, American Samoa, Northern Mariana Islands, Brunei Darussalam, Federal States of Micronesia and Tokelau, majority of which are Pacific Island countries (PICs) except Cambodia.

Table 2 shows public health law coverage by subjects. More than 90% of the countries have public health laws for issues such as health care organisation, communicable diseases, substance abuse, health workers, and environmental protection. Issues with more than 80% and less than 90% coverage were international treaties, smoking controls, mental health, post-mortem examinations, disposal of the dead, and food safety. Issues with low coverage were human reproduction, family health, oral health, medical devices, organ transplantation, and human rights. Only less than 50% of the countries have public health law coverage for such issues. The average by sub-categories on general provision, health system, disease control, healthy community and population, health ethics, and health security was not statistically different (F(t) = 0.43, *p*-value = 0.82).

**Table 2:** Public health law coverage of countries by subjects

Category	Variables	Mean ± SD	(*n* = 33)		
			N (%)		
			Yes	No	DNK
General provision	Constitutional provision on health	21.7 ± 6.7	20 (60.6)	13 (39.4)	0 (0.0)
	Human rights		16 (48.5)	21 (63.6)	2 (6.1)
	International treaties		29 (87.9)	3 (9.1)	1 (3.0)
Health system	Health care organization	22.8 ± 5.1	31 (93.9)	0 (0.0)	2 (6.1)
	Health financing		21 (63.6)	10 (30.3)	2 (6.1)
	Health research		18 (54.5)	11 (33.3)	3 (9.1)
	Health education		23 (60.6)	7 (21.2)	3 (9.1)
	Health workers		30 (90.9)	2 (6.1)	1 (3.0)
	Health care facilities		26 (78.8)	4 (12.1)	3 (9.1)
	Pharmaceuticals		25 (60.6)	8 (24.2)	0 (0.0)
	Traditional medicines		19 (57.6)	12 (36.4)	2 (6.1)
	Medical devices		16 (48.5)	12 (36.8)	5 (15.2)
	Health information		19 (60.6)	9 (27.3)	5 (15.2)
Disease control	Communicable disease	22.3 ± 6.5	31 (93.9)	1 (3.0)	1 (3.0)
	HIV/AIDS		23 (69.7)	10 (30.3)	0 (0.0)
	Organ transplantation		16 (48.5)	13 (39.4)	4 (12.1)
	Non-communicable disease		19 (60.6)	11 (33.3)	3 (9.1)
Healthy community and population	Oral health	20.6 ± 6.8	13 (39.4)	15 (45.5)	5 (15.2)
	Family health		12 (36.4)	16 (48.6)	5 (15.2)
	Child health		24 (60.6)	3 (9.1)	6 (18.2)
	Human reproduction		9 (27.3)	19 (57.6)	5 (15.2)
	Elderly care		19 (57.6)	9 (27.3)	5 (15.2)
	Disable care		20 (60.6)	3 (9.1)	6 (18.2)
	Mental health		27 (81.8)	4 (12.1)	2 (6.1)
	Smoking control		28 (84.4)	5 (15.2)	0 (0.0)
	Alcohol control		25 (60.6)	5 (15.2)	3 (9.1)
	Drug abuse		31 (93.9)	2 (6.1)	0 (0.0)
	Nutrition		17 (51.5)	12 (36.4)	4 (12.1)
	Accident prevention		26 (78.8)	4 (12.1)	3 (9.1)
	Sports and recreation		17 (51.5)	9 (27.3)	7 (21.2)
Health ethics issue	Biomedical ethics	23.5 ± 4.4	18 (54.5)	12 (36.4)	3 (9.1)
	Death and dying		22 (66.7)	7 (21.2)	4 (12.1)
	Post-mortem examinations		27 (81.8)	3 (9.1)	3 (9.1)
	Dead disposal		27 (81.8)	5 (15.2)	1 (3.0)
Health Security	Food safety	24.8 ± 5.3	27 (81.8)	2 (6.1)	4 (12.1)
	Poisons and hazardous substances		26 (78.8)	4 (12.1)	3 (9.1)
	Occupational health and safety		25 (60.6)	7 (21.2)	1 (3.0)
	Environmental protection		30 (90.9)	1 (3.0)	2 (6.1)
	Radiation protection		16 (48.5)	12 (36.4)	5 (15.2)

Table 3 shows differences of public health law coverage by general characteristics of countries. There are no statistical differences on income and poverty levels. Further, there are no differences between small developing island states and others, as well as between sovereign states and, overseas territories and dependent areas. Regarding the legal situation, there are no differences among civil, common, and combination law systems, as well as between groups with or without health provisions in their constitutions.

**Table 3:** Differences of public health law coverage by general characteristics of countries

Variables		Categories	Mean ± SD	F(t)	(*n* = 33)
					*P*-value*
Socioeconomic status	Income	High-income country	30.4 ± 8.6	1.29	0.29
		Upper middle-income country	26.8 ± 8.3		
		Lower middle-income country	24.4 ± 8.9		
	Poverty	Least developed country	22.7 ± 4.7	1.31	0.19
		Non-least developed country	27.8 ± 9.2		
Basic characteristics	Islands nations	Small islands developing states	24.5 ± 7.0	1.91	0.06
		Others	30.2 ± 10.0		
	Sovereign and dependent areas	Sovereign states	28.1 ± 8.2	1.30	0.20
		Overseas territories and dependent areas	23.7 ± 10.0		
Legal situation	Legal system	Civil law	31.6 ± 8.8	1.35	0.27
		Common law	25.8 ± 8.5		
		Combination law	24.0 ± 8.9		
	Health Provision in constitution	Yes	28.0 ± 9.7	0.85	0.39
		No	25.3 ± 7.1		

*p-value level of statistical significance ≤0.05

Table 4 is the main table indicating the association of public health law coverage with the health-related SDGs statistics. If the correlation coefficient is more than 0.4 or less than − 0.4, then we interpret that the two variables have an association. Further, the regression analysis statistical significance indicates the existence of an association between the two variables.

**Table 4:** Relationship between public health law coverage and health-related SDG statistics

Categories	Health Indicators	Mean ± SD	Correlation Coefficient (r)	R2	Beta	P-value*
Life expectancy	Life expectancy at birth (years)	73.6 ± 6.5	**0.47**	0.18	0.39	**0.03**
	Health life expectancy at birth (years)	65.5 ± 5.7	**0.47**	0.18	0.33	**0.04**
SDGs Index	Total SDGs index score	69.4 ± 7.1	0.47	0.13	0.45	0.15
	Health-related SDGs Index	60.3 ± 13.8	**0.43**	0.14	0.24	**0.05**
SDGs 3.1	Maternal mortality ratio (per 100,000 live birth)	73.9 ± 64.1	**−0.53**	0.25	−4.35	**0.01**
	Proportion of births attended by skilled health personnel (%)	91.3 ± 14.9	0.15	−0.02	0.27	0.48
SDGs 3.2	Under-five mortality rate (per 1000 live births)	23.2 ± 17.2	−0.37	0.10	−0.81	0.06
	Neonatal mortality rate (per 1000 live births)	12.0 ± 8.0	**−0.44**	0.16	−0.44	**0.02**
SDGs 3.3	New HIV infections among adults 15–49 years old (per 1000 uninfected population)	0.86 ± 1.26	**0.78**	0.53	−5.88	**0.04**
	TB incidence (per 100,000 population)	148.1 ± 140.5	−0.05	−0.04	−1.11	0.79
	Malaria incidence (per 1000 population at risk)	25.5 ± 42.1	0.51	0.16	−0.11	0.16
	Infants receiving three doses of hepatitis B vaccine (%)	88.0 ± 13.3	0.21	0.02	0.34	0.32
	Reported number of people requiring interventions against NTDs	2,480,194 ±8,544,180	0.12	0.01	126, 180	0.57
SDGs 3.4	Probability of dying from any of CVD, cancer, diabetes, CRD between age 30 and 70 (%)	20.9 ± 8.6	−0.37	0.09	−0.40	0.10
	Suicide mortality rate (per 100,000 population)	11.0 ± 7.3	0.34	0.07	0.32	0.13
SDGs 3.5	Total alcohol per capita (≥15 years of age) consumption (litres of pure alcohol), projected estimates	4.8 ± 3.4	**0.45**	0.17	0.20	**0.02**
SDGs 3.6	Road traffic mortality rate (per 100,000 population)	11.9 ± 7.5	0.08	−0.04	0.08	0.71
SDGs 3.7	Proportion of married or in-union women of reproductive age who have their need for family planning satisfied with modern methods (%)	54.2 ± 13.4	0.52	0.20	0.31	0.07
	Adolescent birth rate (per 1000 women aged 15–19 years)	39.8 ± 28.8	**−0.40**	0.12	−1.49	**0.04**
SDGs 3.8	UHC service coverage index	63.9 ± 13.5	**0.50**	0.21	0.86	**0.02**
	Population with house hold expenditures on health > 10% of total household expenditure or income (%)	5.85 ± 4.08	0.66	0.29	0.54	0.16
	Population with house hold expenditures on health > 25% of total household expenditure or income (%)	1.45 ± 1.40	0.06	−0.25	0.00	0.91
SDGs 3.9	Mortality rate attributed to household and ambient air pollution (per 100,000 population)	51.4 ± 42.9	0.04	−0.08	0.20	0.89
	Mortality rate attributed to exposure to unsafe WASH services (per 100,000 population)	3.8 ± 4.8	−0.50	0.19	−0.29	0.06
	Mortality rate attributed to unintentional poisoning (per 100,000 population)	0.9 ± 0.6	−0.23	0.01	−0.02	0.29
SDGs 3.a	Age-standardized prevalence of tobacco smoking among persons 15 years and older (%)	34. 9 ± 18.0	0.20	−0.02	−0.09	0.41
SDGs 3.b	Diphtheria-tetanus-pertussis (DPTs) immunization coverage among 1-year-olds (%)	88.3 ± 12.8	0.24	0.02	0.40	0.23
	Total net official development assistance to medical research and basic health per capita (constant 2014 US$) by recipient country	6.00 ± 5.2	0.39	0.11	−0.27	0.09
SDGs 3.c	Skilled health professional density (per 10,000 population)	60.9 ± 41.8	0.16	−0.02	0.83	0.45
SDGs 3.d	Average of 13 International Health Regulations core capacity scores	78.3 ± 19.8	**0.54**	0.26	1.35	**0.004**
SDGs 1.a	General government health expenditure as % of general government expenditure	12.4 ± 6.1	0.19	−0.01	0.15	0.36
SDGs 2.2	Prevalence of stunting in children under 5 (%)	21.5 ± 14.3	0.44	0.14	−0.27	0.89
	Prevalence of wasting in children under 5 (%)	5.2 ± 3.7	0.16	−0.05	−0.39	0.57
	Prevalence of overweight in children under 5 (%)	12.0 ± 22.6	0.25	−0.01	0.63	0.36
SDGs 6.1	Proportion of population using improved drinking water sources (%)	88.8 ± 15.6	0.21	0.05	0.44	0.33
SDGs 6.2	Proportion of population using improved sanitation (%)	74.1 ± 26.2	0.39	0.11	1.31	0.06
SDGs 7.1	Proportion of population with primary reliance on clean fuels	55.1 ± 34.8	0.31	0.06	1.35	0.13
SDGs 11.6	Annual mean concentrations of fine particulate matter (PM2.5) in urban areas)	16.7 ± 10.3	0.41	0.11	0.49	0.10
SDGs 13.1	Average death rate due to natural disasters (per 100,000 population)	0.8 ± 1.1	−0.08	−0.05	−0.01	0.72
SDGs 16.1	Mortality rate due to homicide (per 100,000 population)	4.3 ± 3.5	−0.04	−0.05	− 0.02	0.86
	Estimated direct deaths from major conflicts (per 100,000 population)	0.09 ± 0.2	0.08	−0.05	0.00	0.72
SDGs 17.1	Completeness of cause-of-death data (%)	88.8 ± 17.0	−0.22	− 0.04	−0.56	0.46

*p-value level of statistical significance ≤0.05

If the value is statistically significant, it is marked in bold

The correlation coefficients between public health law coverage, life expectancy at birth as well as health life expectancy at birth were both 0.47 (*p* = 0.03 and 0.04 respectively). With respect to SDGs, we could not find any association between public health law coverage and the total SDGs index score; however, there was an association between public health law and health-related SDGs index with a correlation coefficient of 0.43 (*p* = 0.05).

Among the SDG 3 indicators, there are statistically significant variables such as maternal mortality ratio (r = − 0.53, *p* = 0.01), neonatal mortality rate (r = − 0.44, *p* = 0.02), new HIV infections (r = 0.78, *p* = 0.04), total alcohol consumption (r = 0.45, p = 0.02), adolescent birth rate (r = − 0.40, *p* = 0.04), UHC service coverage index (r = 0.50, *p* = 0.02), and IHR average core capacity score (r = 0.54, *p* = 0.004).

This table also shows the association of public health law coverage with health factors in other SDGs that are not included in SDG 3 but affect health such as poverty (SDG 1), hunger (SDG 2), clean water and sanitation (SDG 6), affordable and clean energy (SDG 7), sustainable cities and communities (SDG 11), climate action (SDG 13), peace and justice (SDG 16), and partnerships for the goals (SDG 17). These variables are not statistically significant.

## Discussion

In this paper, we reviewed the public health law situation in the selected 33 Western Pacific countries. Further, we analysed the association of public health law coverage with the health status from health-related SDGs statistics using a multi-group ecological study. The number of public health laws in a given country covering given regions differ widely from country to country and a significant number of gaps in domestic public health law are noted. In addition, this study empirically shows that public health law affects several important health indicators such as life expectancy at birth, neonatal mortality rate, and health-related SDGs index.

With regard to the study results, countries with low public health law coverage were mostly PICs. It is assumed that these countries have comparatively small land and population sizes [22] as well as relatively weak legal and health systems. Further, they mostly have common or combination legal systems [23] which have relatively low public health law coverage than civil law systems. Issues associated with low public health coverage are human reproduction, family health, oral health, medical devices, and organ transplantation. We assumed that these might be minor matters directly affecting life or are issues that are of interest to only a few countries. For example, the regulation of medical devices or organ transplantations exist in countries and regions with medical technology. This may also be explained by political sensitivity, inapplicability, low priority, no law necessary, and no relevant traditional legislations. For example, regarding human rights, some governments may be reluctant to enshrine personal freedoms into law. Regarding patients’ rights, some issues may be covered by existing professional codes of conduct and may not be traditionally legislated [24].

It was assumed that there are differences in public health law coverage by socioeconomic status; however, they were not analysed in the results. It is generally implied that establishing advanced law systems may lead to socioeconomic development [25] and we assumed that high-income countries have better public health law systems than middle income countries [26]. However, the analysis showed there were no differences by income or poverty levels which can be interpreted that public health law coverage can be improved irrespective of the socioeconomic status in countries.

There is association between public health law coverage and health-related SDGs statistics such as life expectancy at birth, health life expectancy at birth, and health-related SDGs index. These results prove that there is a strong relationship between public health law and the health-related SDGs. Life expectancy and health life expectancy at birth are upper level health indicators when health-related SDGs are achieved. However, we could not establish an association between public health law coverage and total SDGs index score which could be an assumption because there are many other indicators not related to health. In contrast, the health-related SDGs index is associated with public health law coverage which assumes that public health law may affect the health-related SDGs.

Among the SDG 3 indicators, maternal mortality ratio, neonatal mortality rate, new HIV infections, total alcohol consumption, adolescent birth rate, UHC service coverage index, and IHR average core capacity score are statistically significant. Most of these indicators are related to UHC indicators especially essential health services [27–29]. This can be interpreted that UHC could be an umbrella for other health targets in SDG 3 [30]. IHR core capacity could also be explained considering domestic law is affected by WHO international regulations [31] which have been applied to all its Member States since it was amended by the World Health Assembly in 2005. The reason why we could not find any association of public health law with health statistics in other SDGs is because their indicators have relatively indirect impact compared to SDG 3 having primary emphasis on health.

There are many concerns with the way laws are currently employed to support health [17]. Public health law is oftentimes developed without regard to existing evidence and expertise, therefore not effectively implemented or enforced [32]. Oftentimes it is poorly designed and not effective in supporting the underlying policy objective or have unintended impacts that are harmful to population health. However, the law enables health sector agencies to apply appropriate public health countermeasures. Governments are increasingly reliant on regulatory strategies to advance SDGs. Many countries’ regulatory systems are weak and face challenges including under-resourcing and capacity gaps [6]. Institutions of public administration are the cornerstones to ensuring successful implementation of the SDGs. These institutions are established and defined by law as well as the rights guaranteed to the population by public health law. Law rarely provides a total solution to any problem and it can almost never work in isolation from health policies. Law is just one more possible intervention system that health professionals may draw upon to promote institutional performance, healthy behaviour, and environments. There is a need to closely examine the relationship between law, governance and health, and countries’ support in order to effectively integrate legal interventions into policy making and strategies to achieve the SDGs.

Meanwhile, there are many strategies for achieving the health-related SDGs. This study did not provide comparisons between various intervention ways such as reinforcing health financing or health workforce. In addition, there was no evidence that the public health law strategy is better than other interventions. However, this research explains the value and effectiveness of public health law in attaining the health-related SDGs, which can be one of the ways for strengthening health systems.

This study has several strengths. Firstly, this is the first research covering the public health law situation in several countries. This provides a broad perspective of the region. There have been case reports on specific health issues on the effects of health legislations [33, 34]; however, academic researches showing the whole picture of public health law situations in countries are very rare. Secondly, data of public health law were collected by country experts who are familiar with their legal and public health systems. If the review and analysis of domestic public health law was carried out by foreigners, there could be misunderstandings or some of the current domestic health legislations would be missing. Thirdly, the study empirically shows how national law can support the global norm or regime in the field of public health. In the health sector, studies linking national and global policies or laws are uncommon [35, 36]. Fourthly, this study is very timely and useful when global society is highly interested on how to achieve the SDGs. Nowadays, one of the most important topics in global health governance is the health-related SDGs. Even though the study has several advantages, there are many weaknesses as well.

Public health law coverage as an independent variable was measured from 2013 to 2016 while health-related SDGs statistics were data from 2005 to 2016. Measurement time is different within both similar and different indicators which makes interpretation difficult. Even though, public health law can theoretically affect the attainment of health-related SDGs in the WHO model [7], the analysis cannot infer causal relationships between two variables while it only provides association between those under the multi-group ecological study design. Multiple regression model is usually used in epidemiological association studies for reducing bias; however, this study used the simple regression analysis because there was no statistical significance among the countries’ general characteristics. There could be potentially unknown confounding factors which may also lead to bias. Further, multi-level models are usually used for investigating ecological effects; however, this study only applied a single level analysis. This is because public health law coverage is both an independent and a global variable significant at the group level.

However, there were compensating efforts for strengthening the design of the multi-group ecological study. The potential strategy is to use groups of smaller sizes in order to reduce confounding risks. The participants in this study were geographically close and the total number of analysed countries is only 33 which is smaller compared to the number of other global nations. Further, 33 out of 37 countries in the Western Pacific Region which is 89.2%, is significant enough for the study to have sufficient statistical power. In addition, there could be a selection bias in this study because we could not analyse the current public health law situation in all the 37 countries in the region.

There are significant amounts of missing data in the health-related SDGs statistics. Especially, the small island countries which have an insignificant amount of SDGs indicators’ data. There are unintended differences within the group which cause selection bias. There could be measurement errors in public health law coverage because many local researchers conducting in-country analysis and definitions of public health law could differ in opinion. For minimizing variations among investigators, we provided assessment guidance, standardised report templates, and offline training courses before the analysis. We also tried to audit the assessment results for quality control.

This is a typical legal health epidemiology study. From this point of view, there are only national level legal health epidemiology studies. This study provides academic meaning in the sense that it works on a global level. However, there is the limitation that it only covers the existence of public health law itself with no knowledge of the law implementation situation. In the legal health epidemiological research, interventional and infrastructural laws are inputs while legal practices, changes in environments and behaviours are mediators, and population health could be the outcome [37]. If we had data on the actual practice of public health law in the countries, a more complete picture could be provided. However, this study still has academic value for future public health law research.

Moreover, there are practical implications of this study. Countries and regions need to focus on enacting or revising their public health laws for better health systems and achieving the SDGs. As a leading global health agency, WHO/WPRO gives technical support to Member States for improving their public health law capacities.

This study showed the importance of broadening public health law coverage but does not elaborate how countries can integrate laws into their overall strategies for managing health systems and achieving population health goals. Consequently, the implementation of public health laws and legal practice need to be reviewed for further study. Assuming that the situation on UHC can be a mediator between public health law and the health-related SDGs, an in-depth study including UHC could be applied. Further, qualitative research such as content analysis of public health law or key informant interviews for real public health law situations could also be considered for a more comprehensive understanding of public health laws in the Western Pacific Region.

## Conclusions

It is clear that public health law is crucial for better governance which is one of the key areas of improving health systems. In addition, resilient health systems support the achievement of the health-related SDGs. In this regard, there is need to focus on public health law.

To the best of our knowledge, this study is the first to review the overall public health law situation in the Western Pacific Region. It also provides strong evidence on the importance of public health law for supporting the attainment of health-related SDGs. This is important in the advancement of the public health law research field.

Strengthening public health law in countries can help in the implementation of the health-related SDGs. These results should ideally be used as the basis for review and action at the country level for improving public health law for better health systems and attainment of health-related SDGs.

## Abbreviations

AIBHL: Asian Institute for Bioethics and Health Law
GBD: Global Burden of Diseases, Injuries, and Risk Factor 2015 study
MDGs: Millennium Development Goals
MOH: Ministry of Health
PICs: Pacific Island countries
SDGs: Sustainable Development Goals
SDSN: Sustainable Development Solution Network
UHC: Universal Health Coverage
WHO: World Health Organization
WHO/WPRO: World Health Organization Western Pacific Regional Office
